# Comparative Antioxidant Potentials and Quantitative Phenolic Compounds Profiles among the Flowers and Leaves from Various *Chrysanthemum morifolium* Cultivars

**DOI:** 10.3390/ph17030340

**Published:** 2024-03-06

**Authors:** Tham Thi Mong Doan, Gia Han Tran, Toan Khac Nguyen, Ki Sung Kang, Jin Hee Lim, Sanghyun Lee

**Affiliations:** 1 Department of Bio-Industry Resources Engineering, Sejong University, Seoul 05006, Republic of Korea; doanthimongtham@gmail.com; 2 Department of Plant Science and Technology, Chung-Ang University, Anseong 17546, Republic of Korea; giahan1121997@gmail.com; 3 Department of Plant Biotechnology, Sejong University, Seoul 05006, Republic of Korea; toanchrys@sju.ac.kr; 4 College of Korean Medicine, Gachon University, Seongnam 13120, Republic of Korea; kkang@gachon.ac.kr; 5 Natural Product Institute of Science and Technology, Anseong 17546, Republic of Korea

**Keywords:** *Chrysanthemum morifolium*, antioxidant, 3,5-dicaffeoylquinic acid, 4,5-dicaffeoylquinic acid

## Abstract

*Chrysanthemum morifolium* is a valuable plant that contains a wide range of phytochemical compounds and exhibits various biological activities. Ethanol extracts from both the leaves and flowers of 17 different cultivars of *C. morifolium* were tested for antioxidant activities using the 2,2-diphenyl-1-picrylhydrazyl and 2,2′-azinobis (3-ethylbenzothiazoline-6-sulfonic acid) assays and were quantitatively analyzed for 12 phenolic compounds using high-performance liquid chromatography with diode-array detection. We found that the ‘Ford’ and ‘Raina’ cultivars demonstrated strong antioxidant abilities and high phenolic compound contents compared to other cultivars, while the flowers of ‘Cielo’ and the leaves of ‘White Cap’ exhibited low antioxidant capacity in both assays. The ‘Cielo’ cultivar also displayed the lowest compound contents. Additionally, in most samples, 3,5-dicaffeoylquinic acid and 4,5-dicaffeoylquinic acid stood out as high-content compounds in the extracts. This study provides foundational knowledge that can be used for selecting appropriate *C. morifolium* cultivars for further research. Moreover, the ‘Ford’ and ‘Raina’ cultivars, containing high amounts of bioactive compounds and showing superior antioxidant ability, could be applied to produce health-beneficial products.

## 1. Introduction

The widespread recognition of the oxidation reaction’s significance to both the human body and food has led to an understanding that oxidative metabolism is vital for cell survival. However, a consequence of this reliance is the generation of free radicals and other reactive oxygen species, resulting in oxidative changes [1,2]. Over the past decade, diseases or disorders associated with oxidative stress—including metabolic, neurodegenerative, cardiovascular, and mitochondrial diseases and cancer—have garnered considerable attention [3,4,5]. Numerous studies have explored the underlying triggers, seeking to elucidate the mechanisms of action of free radicals and identify effective substances that prevent or reverse oxidative damage [6,7].

Antioxidants demonstrate high efficacies in regulating the production of free radicals, preventing their undesirable effects, and supporting the body’s antioxidant and detoxifying mechanisms [8,9,10,11]. They can naturally occur in plants, animals, and microorganisms or may be synthesized through chemical means. Higher plants and their component compounds serve as abundant sources of natural antioxidants, such as tocopherols and polyphenols, which are prevalent in spices, herbs, fruits, vegetables, cereals, grains, seeds, teas, and oils [12,13,14]. The isolation of naturally occurring antioxidants as pure compounds from their source materials opens possibilities for their use in nutraceutical and pharmaceutical applications [15,16]. Recently, numerous methods have been employed to evaluate the efficacy of natural antioxidants. These methods encompass assays such as DPPH (2,2-diphenyl-1-picrylhydrazyl) and ABTS^+^ (2,2-azino-bis-3-ethylbenzothiazoline-6-sulphonic acid), the ferric reducing antioxidant power, the β-carotene/linoleic acid, the Rancimat method, and the inhibition of low-density lipoprotein oxidation. Notably, among these methods, DPPH and ABTS are two popular approaches [17].

Phenolic compounds—widely present in medicinal plants, spices, vegetables, fruits, grains, pulses, and seeds—encompass thousands of molecules with diverse chemical structures. They constitute a significant category of natural antioxidants with potential positive impacts on human health [18]. These compounds have been reported to exhibit various biological activities, including antioxidant activity [19,20,21]. Recently, phenolics have gained recognition as potent antioxidants in vitro, surpassing the antioxidant potencies of vitamins E and C, as well as carotenoids [22,23,24,25]. The inverse relationship between fruit and vegetable intake and the risks of cardiovascular and neurodegenerative diseases, cancer, diabetes, and osteoporosis has been partially attributed to phenolics [26,27,28,29,30]. These benefits have earned the attention of scientists, food manufacturers, and consumers, and this growing interest aligns with the evolving trend toward functional foods designed to offer specific health benefits [31,32,33].

*Chrysanthemum*, a genus in the Asteraceae family, comprises approximately 40 species and is extensively distributed in Asia, notably in Mongolia, China, Japan, and eastern Europe [34]. Though it is predominantly cultivated worldwide for ornamental purposes today [35], *Chrysanthemum morifolium* Ramat is recognized as one of the most economically significant ‘food and drug dual-use’ plants globally and has been traditionally consumed for health and disease prevention [36,37,38]. As people’s living standards continue to rise, and health awareness increases, dietary herbal medicines like *C. morifolium* are gaining greater importance in daily life [39]. Notably, *C. morifolium* is renowned for its traditional efficacies, including scattering cold, brightening eyes, reducing heat, and cleansing toxins, making it applicable for conditions such as swelling and pain of the eyes, common colds with the wind–heat pattern, and dim-sightedness [40]. Additionally, numerous modern pharmacological studies have reported diverse key bioactive compounds in *C. morifolium*, such as phenolics (isochlorogenic acid C, chlorogenic acid, and cryptochlorogenic acid), caffeoylquinic acids, flavonoids (luteolin, apigenin-7-glucoside, and apigenin), and carotenoids, steroids, and terpenoids [41,42]. These components exhibit various activities, displaying anticancer, antimutagenic, anti-inflammatory, anti-human immunodeficiency viruses, antioxidative, neuroprotective, antidiabetic, antihypertensive, and aldose reductase inhibition effects [43,44,45,46].

In this study, the compounds from the flowers and leaves of 17 different *C. morifolium* cultivars were extracted and evaluated for their antioxidant ability using DPPH and ABTS^+^ assays. Subsequently, these samples were analyzed for 12 phenolic compounds using high-performance liquid chromatography with diode-array detection (HPLC-DAD).

## 2. Results

### 2.1. Antioxidant Activity

The antioxidant activities of extracts from the flowers and leaves of different *C. morifolium* cultivars were evaluated based on their IC_50_ values (Table 1 and Table 2).

The higher the IC_50_, the lower the antioxidant ability, and vice versa. In general, the IC_50_ values of the flower samples ranged from 1.26 to 6.21 mg/mL in the DPPH assay and 1.14 to 5.65 mg/mL in the ABTS^+^ assay, while the IC_50_ values of the leaf samples ranged from 2.10 to 20.86 mg/mL in the DPPH assay and 1.82 to 11.48 mg/mL in the ABTS^+^ assay. In contrast, the control sample (ascorbic acid) had an IC_50_ of 0.10 mg/mL, indicating that the antioxidant activity of all samples was more than 10 times lower than that of ascorbic acid. Moreover, in the same cultivar, most flower samples exhibited stronger antioxidant ability than leaf samples. Only in the ‘Yes Holic’ cultivar did leaf samples show a higher antioxidant ability, and this was only true in the DPPH assay; however, the differences between the two IC_50_ values were not remarkable.

According to the DPPH results (Table 1), among the flower samples, the strongest antioxidant activity was found in S3 (‘Powaru’, 1.26 mg/mL), followed by S17 (‘Ford’, 1.27 mg/mL) and then S7 (‘Raina’, 1.35 mg/mL), while the weakest antioxidant activity was found in S13 (‘Cielo’, 6.21 mg/mL). Conversely, the highest antioxidant activity among leaf samples was found in S32 (‘Geumsu’, 2.10 mg/mL), followed by S24 (‘Raina’, 2.18 mg/mL) and S34 (‘Ford’, 2.65 mg/mL), with the lowest antioxidant activity observed in S26 (‘White Cap’, 20.86 mg/mL).

In the ABTS^+^ results (Table 2), S3 (‘Powaru’, 1.14 mg/mL) again demonstrated the highest antioxidant ability among flower samples, followed by S15 (‘Geumsu’, 1.33 mg/mL), S17 (‘Ford’, 1.38 mg/mL), and S7 (‘Raina’, 1.71 mg/mL), while the lowest antioxidant ability was found in S13 (‘Cielo’, 5.65 mg/mL). Similarly, among leaf samples, S32 (‘Geumsu’, 1.82 mg/mL) exhibited the strongest antioxidant ability, followed by S24 (‘Raina’, 1.85 mg/mL), S23 (‘Corsage’, 2.46 mg/mL), and S34 (‘Ford’, 2.59 mg/mL), with the weakest antioxidant ability observed in S26 (‘White Cap’, 11.48 mg/mL).

In summary, among the flower and leaf samples, S3 (‘Powaru’) and S32 (‘Geumsu’) exhibited the highest antioxidant activity, while S13 (‘Cielo’) and S26 (‘White Cap’) demonstrated the lowest. Additionally, among the 17 cultivars, ‘Ford’ and ‘Raina’ stood out as cultivars with high antioxidant levels.

### 2.2. HPLC Analysis

Thirty-four extract samples from *C. morifolium* flowers and leaves were analyzed to quantify the content of 12 phenolic compounds using the HPLC-DAD method.

**Figure 1:**
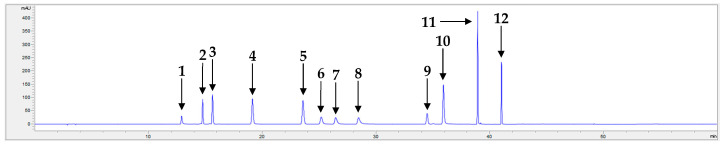
HPLC chromatogram of compounds **1**–**12**: 3-*O*-Caffeoylquinic acid (**1**), schaftoside (**2**), isoschaftoside (**3**), luteolin 7-*O*-glucoside (**4**), apigenin 7-*O*-glucoside (**5**), 3,5-dicaffeoylquinic acid (**6**), 3,4-dicaffeoylquinic acid (**7**), 4,5-dicaffeoylquinic acid (**8**), acacetin 7-*O*-rutinoside (**9**), luteolin (**10**), apigenin (**11**), and acacetin (**12**).

**Figure 2:**
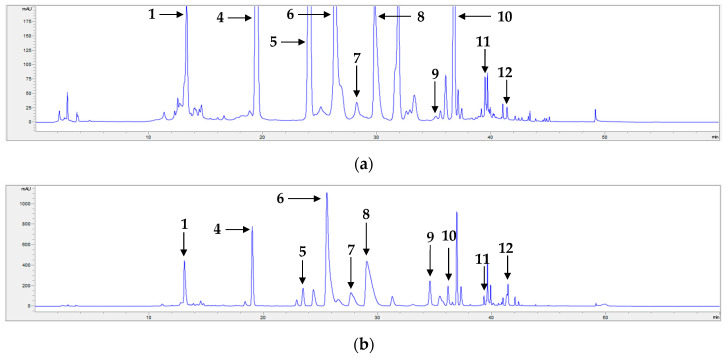
Representative HPLC chromatograms of extracts of the flowers of *C. morifolium* cultivars: (**a**) S3 and (**b**) S17. 3-*O*-Caffeoylquinic acid (**1**), luteolin 7-*O*-glucoside (**4**), apigenin 7-*O*-glucoside (**5**), 3,5-dicaffeoylquinic acid (**6**), 3,4-dicaffeoylquinic acid (**7**), 4,5-dicaffeoylquinic acid (**8**), acacetin 7-*O*-rutinoside (**9**), luteolin (**10**), apigenin (**11**), and acacetin (**12**).

**Figure 3:**
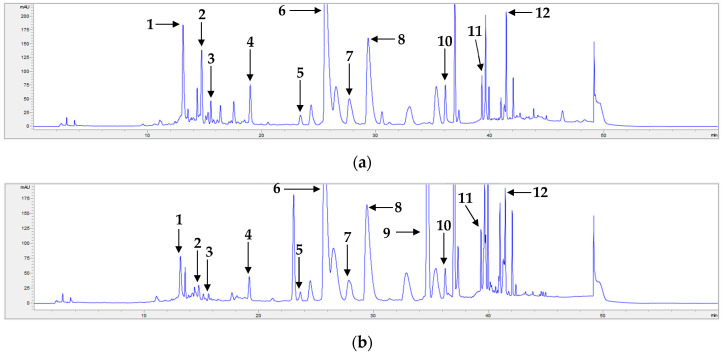
Representative HPLC chromatograms of extracts of the leaves of *C. morifolium* cultivars: (**a**) S24 and (**b**) S32. 3-*O*-Caffeoylquinic acid (**1**), schaftoside (**2**), isoschaftoside (**3**), luteolin 7-*O*-glucoside (**4**), apigenin 7-*O*-glucoside (**5**), 3,5-dicaffeoylquinic acid (**6**), 3,4-dicaffeoylquinic acid (**7**), 4,5-dicaffeoylquinic acid (**8**), acacetin 7-*O*-rutinoside (**9**), luteolin (**10**), apigenin (**11**), and acacetin (**12**).

All compounds were well separated, with retention times spanning from 13.04 to 41.44 min. Additionally, calibration curves for each compound were constructed, and their correlation factors were all 0.9992 or higher (Table 3), demonstrating the excellent linearity of the employed method. Based on these calibration equations, the content of each compound in each sample was determined (Table 4 and Table 5). The chromatograms of standard compounds and the flower and leaf samples are shown in Figure 1, Figure 2 and Figure 3, respectively.

Overall, only four compounds, namely, 3-O-caffeoylquinic acid (**1**), luteolin 7-O-glucoside (**4**), apigenin 7-O-glucoside (**5**), and 4,5-dicaffeoylquinic acid (**8**), were consistently present in all samples. In contrast, schaftoside (**2**) and isoschaftoside (**3**) were detected in only some samples and in relatively low amounts. Luteolin 7-O-glucoside (**4**), 3,5-dicaffeoylquinic acid (**6**), and 4,5-dicaffeoylquinic acid (**8**) contents were generally high in flower samples, whereas most leaf samples showed high levels of 3,5-dicaffeoylquinic acid (**6**) and 4,5-dicaffeoylquinic acid (**8**).

Showing consistency with the antioxidant activity results, the majority of flower samples contained higher concentrations of the surveyed compounds than the leaf samples. Nevertheless, exceptions were noted in the ‘Argus’ and ‘Geumsu’ cultivars, where leaf samples exhibited a slightly higher content.

Concerning the total content of the 12 compounds in the flower extracts, S17 (‘Ford’, 200.68 mg/g extract) exhibited the highest content, followed by S7 (‘Raina’, 149.82 mg/g extract) and then S3 (‘Powaru’, 120.75 mg/g extract), with S13 (‘Cielo’, 26.32 mg/g extract) displaying the lowest content. However, in leaf extracts, S32 (‘Geumsu’, 81.73 mg/g extract) exhibited the highest content, followed by S24 (‘Raina’, 64.95 mg/g extract), S18 (‘Argus’, 41.16 mg/g extract), S20 (‘Powaru’, 38.94 mg/g extract), and S34 (‘Ford’, 35.69 mg/g extract) while the lowest content was observed in S26 (‘White Cap’, 5.52 mg/g extract) and S30 (‘Cielo’, 4.75 mg/g extract). 

Taken together, S17 (‘Ford’) and S32 (‘Geumsu’) exhibited the highest total compound contents in flowers and leaves, respectively, whereas flower sample S13 (‘Cielo’) and leaf samples S26 (‘White Cap’) and S30 (‘Cielo’) displayed the lowest. The ‘Ford’ and ‘Raina’ cultivars generally showed the highest total compound contents among the cultivars, while ‘Cielo’ showed the lowest.

## 3. Discussion

Phenolic compounds, comprising flavonoids and phenolic acids, are recognized as contributors to the antioxidant capacities of fruits. Additionally, fruits containing higher phenolic contents typically exhibit stronger antioxidant capacities [47,48]. This trend was also observed in this study, where the levels of 12 phenolic compounds in the *C. morifolium* extracts were closely related to their antioxidant capacity. Specifically, flower extract samples S17, S7, and S3, which contained high compound levels, exhibited stronger antioxidant capacities than other samples. Conversely, S13, with a low compound level, showed the weakest antioxidant ability among the flower samples. A similar trend was observed in leaf samples, where high-compound content samples S32, S24, and S34 demonstrated strong antioxidant activity, while S26, with a low compound content, exhibited weak antioxidant capacity.

In contrast, some samples exhibited high levels of phenolic compounds, yet their antioxidant activity was notably low. It is crucial to note that this study exclusively focused on 12 phenolic compounds. Many other compounds present in the samples were not evaluated. Previous studies have identified a wide range of bioactive compounds in *C. morifolium*, including quercetin, isorhamnetin 3-*O*-glucoside, eriodictyol, pyracanthoside, apigetrin, acacipetalin, diosmetin, spinacetin, axillarin, bonanzin, cirsiliol, chrysosplenol D, artemetin, quercitrin, acacetin 7-*O*-glucoside, apigenin 7-*O*-glucoside, and luteolin 7-*O*-glucoside [49,50,51]. Therefore, the results of this study only partially characterize the samples, and further research is necessary to conclusively identify which compounds significantly influence the antioxidant capacity of *C. morifolium* extracts. Additionally, it is important to note that many other compounds, besides phenolic compounds, contribute to the biological activities of *C. morifolium*, such as carotenoids, steroids, and terpenoids. Therefore, the presence of phenolic compounds contributes to the antioxidant ability, but it cannot be said that the antioxidant ability as well as biological processes are solely determined by phenolic compounds.

On the other hand, while the antioxidant ability of the flower samples was considerably higher than that of the leaf samples, it is easier to cultivate and obtain a high amount of leaf samples rather than flower samples. Furthermore, in contrast to leaf samples, extracting flower samples is more prone to obtaining undesirable substances such as oils. Consequently, it is essential to carefully consider both antioxidant abilities and other relevant conditions when choosing the appropriate samples.

In our previous study, we quantified the antioxidant capacity as well as the content of phenolic compounds (including the 12 compounds this study focused on) in three different *Chrysanthemum* species from various regions in South Korea [52]. The IC_50_ values of the extracts in that study ranged from 5.8 to 17.4 mg/mL in a DPPH assay and 2.7 to 9.4 mg/mL in an ABTS^+^ assay, and the total content of phenolic compounds ranged from 7.87 to 95.13 mg/g extract. In the current study, the flower extracts exhibited IC_50_ values ranging from 1.14 to 6.21 mg/mL in the DPPH assay and 1.14 to 5.65 mg/mL in an ABTS^+^ assay, and the total content of phenolic compounds ranged from 26.32 to 200.68 mg/g extract. Thus, both the antioxidant capacity and the bioactive compound levels in the flower samples from the 17 cultivars in this study surpassed those observed in the previous study despite similar extraction procedures. 

Another study reported that *C. zawadskii* var. *lucidum* flowers, containing high levels of 3-*O*-caffeoylquinic acid, 3,5-dicaffeoylquinic acid, 4,5-dicaffeoylquinic acid, and acacetin 7-*O*-rutinoside, exhibited skin-whitening activity by inhibiting melanin production in α-melanocyte-stimulating hormone-induced B16F10 cells [53,54,55]. The study also confirmed their antimicrobial activity against *Staphylococcus aureus*, *Pseudomonas aeruginosa*, and *Candida albicans*. Therefore, the samples in this study with high concentrations of phenolic compounds are also expected to demonstrate both skin-whitening and antimicrobial activities.

Furthermore, most previous studies have concentrated on investigating compounds and biological activities in the flowers of *Chrysanthemum* species rather than the leaves. Therefore, our study not only demonstrates the differences in biological properties between the flowers and leaves of *Chrysanthemum* species but also fills the gap in studies on the leaves of *Chrysanthemum* species. Although the biological activity of the leaves is not as potent as that of the flowers, it is evident that *C. morifolium* leaves still exhibit considerable biological activity that warrants further investigation.

## 4. Materials and Methods

### 4.1. Plant Materials

**Figure 4:**
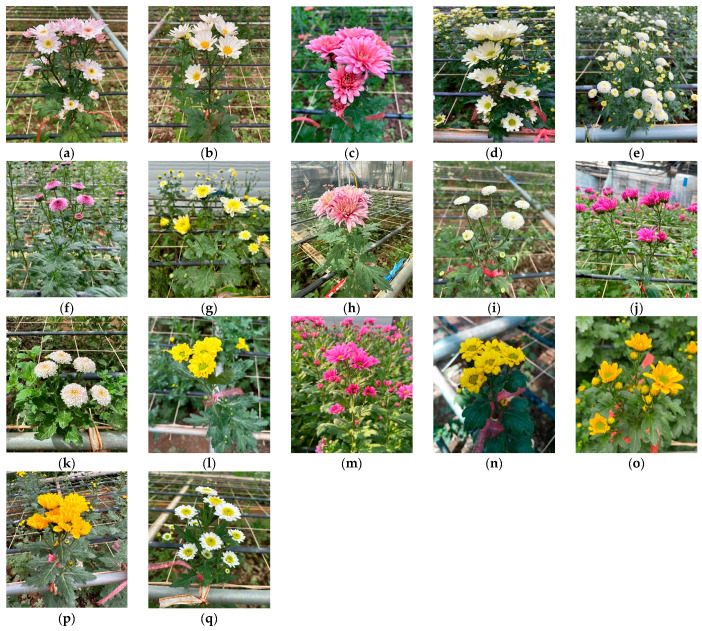
*C. morifolium* cultivars used in this study: (**a**) ‘Argus’, (**b**) ‘Young cream’, (**c**) ‘Powaru’, (**d**) ‘Fire Cream’, (**e**) ‘Argento’, (**f**) ‘Corsage’, (**g**) ‘Raina’, (**h**)’ Coral King’, (**i**) ‘White Cap’, (**j**) ‘Glorious Days’, (**k**) ‘Cutie Bubble’, (**l**) ‘Melina’, (**m**) ‘Cielo’, (**n**) ‘Shingeumsu’, (**o**) ‘Geumsu’, (**p**) ‘Yes Holic’, and (**q**) ‘Ford’.

Seventeen cultivars of *C. morifolium* (Figure 4) were grown by Prof. Jinhee Lim, Sejong University, Republic of Korea, in May 2023. 

Seedlings were sown in May 2023, and the leaves and flowers were freshly harvested in September 2023 (~16 weeks old), dried, and cut into small pieces before extraction. All specimens (S1–S34) were deposited at the herbarium of the Department of Bio-Industry Resources Engineering, Sejong University, Seoul, Republic of Korea.

### 4.2. Instruments and Reagents

The HPLC analysis was conducted using an Agilent 1260 Infinity II Quat Pump (Santa Clara, CA, USA) and a DAD. The configuration comprised a pump and an auto-sampler, integrated with a YMC Pack Pro C18 column (4.6 × 250 mm, 5 µm). The HPLC-grade solvents, including water, acetonitrile, and methanol (MeOH), were procured from J. T. Baker (Philipsburg, PA, USA). In addition, acetic acid was acquired from Samchun Chemicals (Pyeongtaek, Republic of Korea). In the context of the assays, both an Epoch microplate spectrophotometer from BioTek (Winooski, VT, USA) and a microplate reader were utilized.

**Figure 5:**
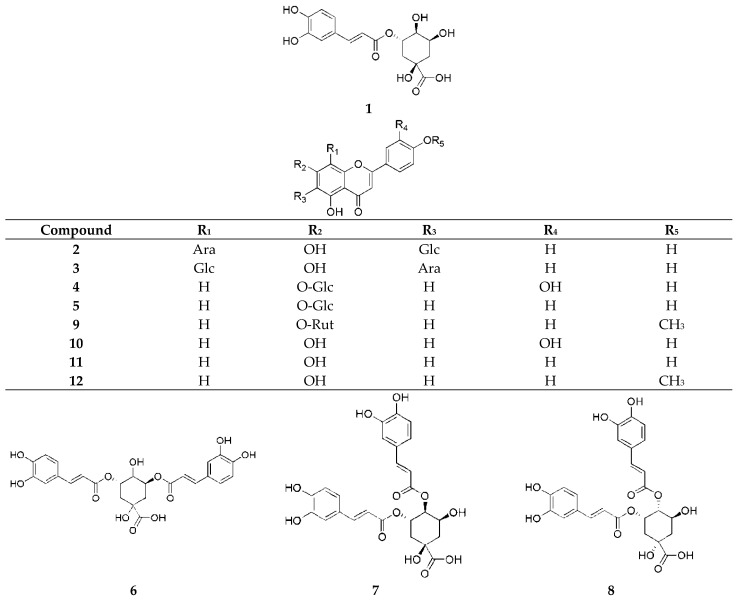
Chemical structures of 3-*O*-caffeoylquinic acid (**1**), schaftoside (**2**), isoschaftoside (**3**), luteolin 7-*O*-glucoside (**4**), apigenin 7-*O*-glucoside (**5**), 3,5-dicaffeoylquinic acid (**6**), 3,4-dicaffeoylquinic acid (**7**), 4,5-dicaffeoylquinic acid (**8**), acacetin 7-*O*-rutinoside (**9**), luteolin (**10**), apigenin (**11**), and acacetin (**12**).

To determine radical scavenging activity, DPPH and ABTS^+^ assays were performed using potassium persulfate obtained from Sigma (Burlington, MA, USA). Additionally, a collection of 12 phenolic compounds (Figure 5) was sourced from the Natural Product Institute of Science and Technology (www.nist.re.kr (accessed on 7 July 2023)), Anseong, Republic of Korea. These were assigned numbers from **1** to **12** for convenience throughout the paper and included 3-*O*-caffeoylquinic acid (**1**), schaftoside (**2**), isoschaftoside (**3**), luteolin 7-*O*-glucoside (**4**), apigenin 7-*O*-glucoside (**5**), 3,5-dicaffeoylquinic acid (**6**), 3,4-dicaffeoylquinic acid (**7**), 4,5-dicaffeoylquinic acid (**8**), acacetin 7-*O*-rutinoside (**9**), luteolin (**10**), apigenin (**11**), and acacetin (**12**).

### 4.3. Sample Extraction and Preparation

For each *C. morifolium* cultivar, 5 g each of dried flower and leaf tissue was subjected to extraction using ethanol in a reflux extractor for 3 h. This extraction process was replicated three times. Following dehydration in a rotary evaporator, the extracts were gathered. For each extract, 20 mg was precisely measured and diluted in 1 mL of MeOH or 1 mL of distilled water to form stock solutions for the DPPH and ABTS^+^ assays, respectively. After filtering through a 0.45 µm membrane filter, sequential dilutions were performed on the stock solutions. To prepare for the HPLC analysis, each extract was dissolved in MeOH, appropriately diluted, and formulated. After dissolution by ultra-sonication, the solution was filtered using a 0.45 μm polyvinylidene fluoride (PVDF) membrane filter to prepare the test solution. For each of the 12 standard compounds, 4 mg was precisely weighed and dissolved in 1 mL of MeOH to create a 4000 ppm stock solution. After complete dissolution by ultra-sonication, the solutions were filtered using a 0.45 μm PVDF membrane filter.

### 4.4. DPPH Radical Scavenging Activity

The DPPH radical scavenging assays were performed using 0.2 mM DPPH working solutions created by diluting the original DPPH stock solution with MeOH. Next, 10 µL of each test solution was combined with 200 µL of the DPPH working solution in respective wells of a 96-well plate. This was repeated three times to ensure accuracy. Then, the solutions were mixed thoroughly using a microplate shaker and incubated in darkness for 30 min. Subsequently, the solution’s absorbance was measured at a wavelength of 514 nm, and the DPPH radical scavenging rate was calculated. The calculated rates were used to construct activity concentration curves for determining the IC_50_ values.

### 4.5. ABTS^+^ Radical Scavenging Activity

The ABTS^+^ radical scavenging assay was performed by diluting the ABTS^+^ stock solution in water to create the ABTS^+^ working solutions. Subsequently, 10 µL of test solution was combined with 200 µL of the ABTS^+^ working solution in a 96-well plate, with the reaction being replicated three times. The solutions were mixed thoroughly on a microplate shaker and incubated for 30 min in the dark before the absorbance was measured at 734 nm. The ABTS^+^ radical scavenging rates were calculated and used to construct curves for IC_50_ determination.

### 4.6. HPLC Conditions

A quantitative chemical analysis of the extracts was conducted using a reverse-phase HPLC system, employing a YMC Pack-Pro C18 column (25 cm × 4.6 mm, 5 μm), and a gradient elution. The mobile phase was composed of 0.25% acetic acid in water (A) and acetonitrile (B), and the elution conditions were 10% B from 0 min to 5 min, increasing to 20% B at 10 min, 27% B at 30 min, 40% B at 35 min, and 100% B at 40 min, which was maintained until 60 min. The column temperature was retained at 30 °C, with an injection volume of 10 μL, a flow rate of 1.0 mL/min, and wavelength monitoring set to 356 nm.

### 4.7. Calibration Curve

The calibration curve for the HPLC-DAD analysis was generated by plotting the concentrations of the 12 compounds’ standard solutions against their corresponding peak areas. To evaluate the linearity of the curves, we used the correlation coefficient (*r*^2^), and then the calibration curves were used to compute the concentrations of the standard compounds in the samples. The calibration equations were established using the mean peak area value (Y) ± standard deviation (n = 3) and concentration (X, µg/mL).

## 5. Conclusions

The antioxidant activities of the flower and leaf extracts from 17 cultivars of *C. morifolium* were evaluated using DPPH and ABTS^+^ assays. Additionally, the presence of 12 phenolic compounds in the extract samples was detected and quantified through HPLC-DAD analysis. The results revealed that 2 out of the 17 cultivars (‘Ford’ and ‘Raina’) exhibited particularly strong antioxidant activities and particularly high compound contents. Moreover, 3,5-dicaffeoylquinic acid (**6**) and 4,5-dicaffeoylquinic acid (**8**) exhibited high contents relative to the other compounds in most of the samples. This study contributes valuable insights into the compounds that various cultivars of *C. morifolium* can offer and also adds to research on other *Chrysanthemum* species. Given the high content of bioactive compounds and the remarkable antioxidant capacity observed in the ‘Ford’ and ‘Raina’ cultivars, they can be considered potential sources of natural materials applicable in diverse industries, including pharmaceuticals, cosmetics, and food.

## Figures and Tables

**Table 1:** DPPH and ABTS^+^ radical scavenging activities of extracts from the flowers of *C. morifolium* cultivars.

Cultivar	IC_50_ (mg/mL)	
	DPPH	ABTS^+^
‘Argus’ flower (S1)	2.83 ± 0.13	2.64 ± 0.25
‘Young cream’ flower (S2)	2.59 ± 0.15	2.40 ± 0.14
‘Powaru’ flower (S3)	1.26 ± 0.08	1.14 ± 0.11
‘Fire Cream’ flower (S4)	1.93 ± 0.19	2.02 ± 0.01
‘Argento’ flower (S5)	2.68 ± 013	2.94 ± 0.07
‘Corsage’ flower (S6)	1.72 ± 0.14	1.77 ± 0.14
‘Raina’ flower (S7)	1.35 ± 0.06	1.71 ± 0.05
‘Coral King’ flower (S8)	1.99 ± 0.10	2.44 ± 0.32
‘White Cap’ flower (S9)	2.53 ± 0.19	2.66 ± 0.29
‘Glorious Days’ flower (S10)	2.64 ± 0.07	2.45 ± 0.36
‘Cutie Bubble’ flower (S11)	1.45 ± 0.05	1.92 ± 0.06
‘Melina’ flower (S12)	4.12 ± 0.39	4.49 ± 0.64
‘Cielo’ flower (S13)	6.21 ± 0.47	5.65 ± 0.49
‘Shingeumsu’ flower (S14)	2.61 ± 0.27	2.45 ± 0.29
‘Geumsu’ flower (S15)	1.74 ± 0.06	1.33 ± 0.07
‘Yes Holic’ flower (S16)	3.64 ± 0.13	3.16 ± 0.19
‘Ford’ flower (S17)	1.27 ± 0.06	1.38 ± 0.09
Ascorbic acid	0.10 ± 0.00	0.10 ± 0.00

Ascorbic acid was employed as the standard and as a reference.

**Table 2:** DPPH and ABTS^+^ radical scavenging activities of extracts from the leaves of *C. morifolium* cultivars.

Cultivar	IC_50_ (mg/mL)	
	DPPH	ABTS^+^
‘Argus’ leaf (S18)	3.21 ± 0.18	3.17 ± 0.21
‘Young cream’ leaf (S19)	6.45 ± 0.62	4.96 ± 0.19
‘Powaru’ leaf (S20)	2.84 ± 0.24	2.95 ± 0.13
‘Fire Cream’ leaf (S21)	3.32 ± 0.23	3.53 ± 0.04
‘Argento’ leaf (S22)	4.74 ± 0.21	3.59 ± 0.07
‘Corsage’ leaf (S23)	3.21 ± 0.19	2.46 ± 0.04
‘Raina’ leaf (S24)	2.18 ± 0.19	1.85 ± 0.10
‘Coral King’ leaf (S25)	5.32 ± 0.28	3.89 ± 0.11
‘White Cap’ leaf (S26)	20.86 ± 0.91	11.48 ± 0.26
‘Glorious Days’ leaf (S27)	4.33 ± 0.26	3.66 ± 0.25
‘Cutie Bubble’ leaf (S28)	7.15 ± 0.16	6.36 ± 0.05
‘Melina’ leaf (S29)	17.25 ± 0.34	9.48 ± 0.37
‘Cielo’ leaf (S30)	6.29 ± 0.38	6.29 ± 0.30
‘Shingeumsu’ leaf (S31)	15.18 ± 0.72	8.24 ± 0.16
‘Geumsu’ leaf (S32)	2.10 ± 0.09	1.82 ± 0.11
‘Yes Holic’ leaf (S33)	3.40 ± 0.07	3.47 ± 0.07
‘Ford’ leaf (S34)	2.65 ± 0.03	2.59 ± 0.11
Ascorbic acid	0.10 ± 0.00	0.10 ± 0.00

Ascorbic acid was employed as the standard and as a reference.

**Table 3:** Calibration curves for compounds **1**–**12**.

Compound	t_R_	Calibration Equation	Correlation Factor, *r*^2^
**1**	13.04	Y = 6.5232X − 76.265	0.9996
**2**	14.81	Y = 10.639X − 26.417	0.9996
**3**	15.54	Y = 16.882X − 29.628	0.9994
**4**	18.80	Y = 19.962X − 97.229	1.0000
**5**	23.26	Y = 18.559X − 15.259	1.0000
**6**	25.22	Y = 7.9331X − 149.49	0.9998
**7**	27.86	Y = 9.2577X − 65.505	0.9992
**8**	28.84	Y = 8.4014X − 173.62	0.9992
**9**	34.63	Y = 9.2925X + 12.17	0.9998
**10**	36.25	Y = 25.5X − 93.216	0.9997
**11**	39.30	Y = 46.97X + 47.153	0.9997
**12**	41.44	Y = 21.014X + 41.394	0.9999

3-*O*-Caffeoylquinic acid (**1**), schaftoside (**2**), isoschaftoside (**3**), luteolin 7-O-glucoside (**4**), apigenin 7-*O*-glucoside (**5**), 3,5-dicaffeoylquinic acid (**6**), 3,4-dicaffeoylquinic acid (**7**), 4,5-dicaffeoylquinic acid (**8**), acacetin 7-*O*-rutinoside (**9**), luteolin (**10**), apigenin (**11**), and acacetin (**12**). t_R_ = retention time; Y = peak area; X = concentration of standards (µg/mL); *r*^2^ = correlation coefficient of five calibration data points (n = 3).

**Table 4:** Contents of compounds **1**–**12** in the flowers of *C. morifolium* cultivars.

Cultivar	Content (mg/g Extract)												
	1	2	3	4	5	6	7	8	9	10	11	12	Total
S1	3.08 ± 0.20	0.18 ± 0.01	ND	5.01 ± 0.27	5.84 ± 0.33	9.28 ± 0.45	1.64 ± 0.09	9.33 ± 0.57	ND	4.17 ± 0.08	2.20 ± 0.01	0.02 ± 0.01	40.76
S2	5.95 ± 0.02	0.16 ± 0.00	ND	9.96 ± 0.12	2.10 ± 0.01	13.38 ± 0.35	1.28 ± 0.02	11.18 ± 0.20	ND	3.51 ± 0.01	0.09 ± 0.00	ND	47.62
S3	11.43 ± 0.43	ND	ND	34.22 ± 0.37	24.23 ± 0.16	23.00 ± 0.21	1.91 ± 0.06	19.84 ± 0.44	0.25 ± 0.02	5.66 ± 0.02	0.12 ± 0.00	0.09 ± 0.00	120.75
S4	7.68 ± 0.09	ND	ND	23.07 ± 0.40	28.47 ± 0.21	15.85 ± 1.06	2.34 ± 0.08	15.12 ± 0.34	ND	5.80 ± 0.05	0.10 ± 0.01	0.08 ± 0.01	98.51
S5	11.03 ± 0.37	ND	ND	9.31 ± 0.12	4.62 ± 0.05	24.83 ± 0.84	4.86 ± 0.21	21.65 ± 0.60	1.90 ± 0.01	1.09 ± 0.02	tr	ND	79.29
S6	8.95 ± 0.21	ND	ND	17.48 ± 0.08	7.91 ± 0.04	11.79 ± 0.34	2.07 ± 0.01	13.05 ± 0.22	2.02 ± 0.07	ND	tr	0.19 ± 0.00	63.46
S7	19.39 ± 0.28	0.61 ± 0.00	tr	9.33 ± 0.08	18.35 ± 0.06	54.72 ± 1.09	4.58 ± 0.06	40.31 ± 0.12	ND	0.44 ± 0.02	0.16 ± 0.00	1.93 ± 0.02	149.82
S8	4.92 ± 0.17	ND	ND	25.56 ± 1.21	22.95 ± 1.04	12.28 ± 0.37	2.58 ± 0.03	13.04 ± 0.31	1.00 ± 0.01	1.77 ± 0.10	0.69 ± 0.01	ND	84.78
S9	4.23 ± 0.04	0.34 ± 0.01	ND	13.15 ± 0.07	4.52 ± 0.02	5.36 ± 0.20	3.07 ± 0.11	21.26 ± 0.16	5.65 ± 0.06	1.16 ± 0.02	0.21 ± 0.00	0.44 ± 0.00	59.39
S10	5.45 ± 0.13	0.20 ± 0.01	ND	13.90 ± 0.11	8.44 ± 0.06	11.43 ± 0.13	2.69 ± 0.09	18.23 ± 0.19	ND	16.03 ± 0.18	1.68 ± 0.00	tr	78.04
S11	11.69 ± 0.29	ND	ND	11.51 ± 0.10	14.82 ± 0.06	17.99 ± 0.28	5.62 ± 0.23	51.05 ± 1.66	ND	0.62 ± 0.02	0.20 ± 0.02	0.56 ± 0.02	114.05
S12	3.43 ± 0.03	0.29 ± 0.01	ND	5.33 ± 0.04	4.47 ± 0.02	3.31 ± 0.30	1.22 ± 0.03	6.13 ± 0.14	8.88 ± 0.07	ND	0.27 ± 0.00	1.05 ± 0.02	34.39
S13	2.21 ± 0.03	0.23 ± 0.01	tr	9.26 ± 0.10	2.39 ± 0.04	2.60 ± 0.09	0.58 ± 0.02	2.41 ± 0.03	0.32 ± 0.02	5.69 ± 0.09	0.57 ± 0.01	0.07 ± 0.01	26.32
S14	7.61 ± 0.14	ND	ND	6.28 ± 0.04	5.77 ± 0.03	8.75 ± 0.58	4.02 ± 0.17	22.87 ± 0.23	5.40 ± 0.07	2.70 ± 0.01	0.14 ± 0.00	0.08 ± 0.01	63.61
S15	6.67 ± 0.30	ND	ND	6.93 ± 0.13	5.72 ± 0.16	8.67 ± 0.52	2.07 ± 0.05	23.95 ± 0.41	14.15 ± 0.13	1.48 ± 0.11	0.32 ± 0.01	0.61 ± 0.02	70.58
S16	4.14 ± 0.11	ND	ND	7.12 ± 0.14	1.20 ± 0.02	6.58 ± 0.13	1.90 ± 0.09	10.45 ± 0.26	ND	2.92 ± 0.05	0.11 ± 0.01	0.15 ± 0.01	34.57
S17	20.07 ± 0.63	ND	ND	11.20 ± 0.04	3.33 ± 0.05	82.93 ± 0.96	11.08 ± 0.07	61.06 ± 0.38	8.04 ± 0.15	2.07 ± 0.02	0.21 ± 0.00	0.69 ± 0.04	200.68

tr = trace; ND = not detected.

**Table 5:** Contents of compounds **1**–**12** in the leaves of *C. morifolium* cultivars.

Cultivar	Content (mg/g Extract)												
	1	2	3	4	5	6	7	8	9	10	11	12	Total
S18	4.53 ± 0.05	0.30 ± 0.01	tr	0.93 ± 0.01	0.26 ± 0.01	21.05 ± 0.26	2.05 ± 0.02	10.71 ± 0.04	0.41 ± 0.01	0.34 ± 0.00	0.59 ± 0.00	tr	41.16
S19	2.56 ± 0.04	0.76 ± 0.01	tr	0.98 ± 0.01	0.21 ± 0.00	11.92 ± 0.19	0.53 ± 0.00	4.45 ± 0.02	ND	1.88 ± 0.02	1.00 ± 0.01	0.35 ± 0.00	24.66
S20	5.80 ± 0.25	tr	ND	0.48 ± 0.00	0.22 ± 0.00	19.78 ± 0.54	1.87 ± 0.03	9.74 ± 0.08	ND	0.77 ± 0.01	0.27 ± 0.00	tr	38.94
S21	4.25 ± 0.08	tr	ND	0.47 ± 0.00	1.29 ± 0.01	15.05 ± 0.20	1.15 ± 0.02	6.50 ± 0.11	0.28 ± 0.01	0.32 ± 0.00	tr	0.07 ± 0.00	29.38
S22	1.57 ± 0.03	tr	ND	0.32 ± 0.01	0.19 ± 0.01	7.88 ± 0.29	0.63 ± 0.01	3.38 ± 0.02	0.98 ± 0.02	tr	tr	0.43 ± 0.01	15.38
S23	6.85 ± 0.27	0.68 ± 0.02	tr	0.34 ± 0.00	0.17 ± 0.01	17.29 ± 0.36	0.69 ± 0.01	7.46 ± 0.11	0.67 ± 0.02	0.19 ± 0.01	tr	0.38 ± 0.01	34.73
S24	7.44 ± 0.22	2.89 ± 0.06	0.51 ± 0.01	1.29 ± 0.01	0.42 ± 0.00	30.57 ± 0.44	3.84 ± 0.06	15.90 ± 0.29	ND	0.89 ± 0.01	0.18 ± 0.00	1.02 ± 0.01	64.95
S25	1.37 ± 0.04	0.15 ± 0.00	tr	1.50 ± 0.01	0.34 ± 0.00	1.81 ± 0.02	0.70 ± 0.02	3.71 ± 0.02	0.41 ± 0.01	3.45 ± 0.02	0.54 ± 0.00	tr	13.98
S26	tr	0.60 ± 0.00	tr	0.39 ± 0.00	tr	ND	tr	1.07 ± 0.01	3.16 ± 0.01	0.21 ± 0.00	tr	0.09 ± 0.00	5.52
S27	1.60 ± 0.01	0.53 ± 0.02	tr	0.90 ± 0.01	tr	2.27 ± 0.04	0.49 ± 0.01	4.08 ± 0.06	ND	0.46 ± 0.00	tr	0.09 ± 0.00	10.42
S28	1.85 ± 0.02	0.15 ± 0.00	ND	0.42 ± 0.00	0.17 ± 0.00	1.10 ± 0.02	0.57 ± 0.01	3.55 ± 0.01	ND	0.19 ± 0.00	tr	0.24 ± 0.00	8.24
S29	tr	0.26 ± 0.01	tr	0.34 ± 0.01	tr	1.80 ± 0.06	ND	1.06 ± 0.00	2.95 ± 0.08	ND	ND	0.03 ± 0.00	6.44
S30	0.73 ± 0.02	0.34 ± 0.01	tr	0.58 ± 0.00	tr	1.03 ± 0.01	ND	1.54 ± 0.01	ND	0.32 ± 0.00	tr	0.20 ± 0.00	4.75
S31	0.76 ± 0.01	0.16 ± 0.01	ND	0.27 ± 0.00	tr	0.93 ± 0.03	tr	1.19 ± 0.00	2.39 ± 0.00	tr	tr	tr	5.69
S32	3.29 ± 0.04	0.53 ± 0.01	tr	0.71 ± 0.01	0.33 ± 0.01	28.23 ± 0.66	3.07 ± 0.03	18.98 ± 0.17	25.00 ± 0.28	0.59 ± 0.01	0.25 ± 0.01	0.76 ± 0.02	81.73
S33	2.72 ± 0.01	0.16 ± 0.01	ND	0.70 ± 0.01	0.20 ± 0.01	4.83 ± 0.10	1.25 ± 0.02	7.02 ± 0.04	tr	1.23 ± 0.01	0.19 ± 0.00	0.75 ± 0.00	19.04
S34	5.87 ± 0.29	1.55 ± 0.04	tr	0.78 ± 0.01	0.22 ± 0.00	5.86 ± 0.33	1.64 ± 0.10	12.96 ± 0.19	6.43 ± 0.04	0.23 ± 0.00	tr	0.14 ± 0.00	35.69

tr = trace; ND = not detected.

## Data Availability

Data are contained within the article.
